# The Dorsal Part of the Anterior Tuberal Nucleus Responds to Auditory Stimulation in Zebrafish (*Danio rerio*)

**DOI:** 10.1523/ENEURO.0062-24.2024

**Published:** 2024-07-09

**Authors:** Carlos Daniel Corrales Parada, Uwe Mayer, Boris P. Chagnaud

**Affiliations:** ^1^Institute for Biology, Karl-Franzens-University Graz, Graz 8010 ST, Austria; ^2^Center for Mind/Brain Sciences, University of Trento, Rovereto 38068 TN, Italy

**Keywords:** anterior tuberal nucleus, auditory processing, indirect marker of neural activity, pS6 time course, teleost fish, torus semicircularis

## Abstract

The zebrafish, a widely used model in neurobiology, relies on hearing in aquatic environments. Unfortunately, its auditory pathways have mainly been studied in larvae. In this study, we examined the involvement of the anterior tuberal nucleus (AT) in auditory processing in adult zebrafish. Our tract-tracing experiments revealed that the dorsal subdivision of AT is strongly bidirectionally connected to the central nucleus of the torus semicircularis (TSc), a major auditory nucleus in fishes. Immunohistochemical visualization of the ribosomal protein S6 (pS6) phosphorylation to map neural activity in response to auditory stimulation substantiated this finding: the dorsal but not the ventral part of AT responded strongly to auditory stimulation. A similar response to auditory stimulation was present in the TSc but not in the nucleus isthmi, a visual region, which we used as a control for testing if the pS6 activation was specific to the auditory stimulation. We also measured the time course of pS6 phosphorylation, which was previously unreported in teleost fish. After auditory stimulation, we found that pS6 phosphorylation peaked between 100 and 130 min and returned to baseline levels after 190 min. This information will be valuable for the design of future pS6 experiments. Our results suggest an anatomical and functional subdivision of AT, where only the dorsal part connects to the auditory network and processes auditory information.

## Graphical abstract

**Figure:**
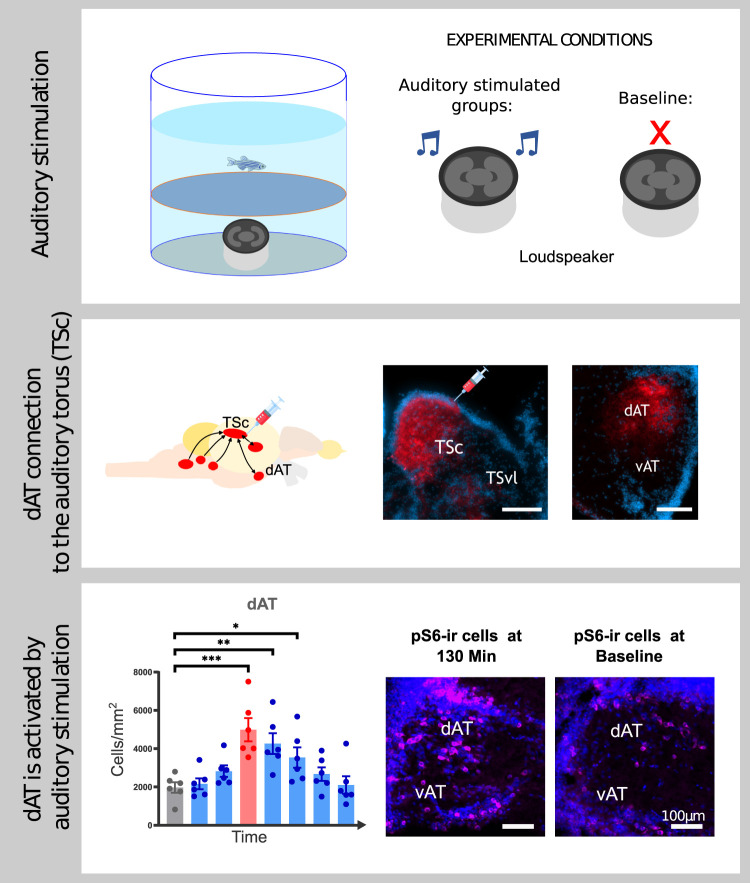
Visual Overview

## Significant Statement

We investigated the involvement of the anterior tuberal nucleus (AT) in zebrafish in auditory processing. Our study revealed a functional and anatomical subdivision of this region. We show that its dorsal subdivision is strongly connected to the central nucleus of the torus semicircularis, a major auditory nucleus in fishes. pS6 phosphorylation, as an indirect marker of neuronal activity after auditory stimulation, substantiated that only the dorsal AT processes auditory information. We also show that after auditory stimulation, pS6 phosphorylation peaked between 100 and 130 min and returned to baseline levels after 190 min, providing valuable information for future studies.

## Introduction

Detecting acoustic stimuli is a crucial sensory ability that allows animals to gain information about the environment. The detection and interpretation of sound source location and spectral composition require complex auditory pathways (Elliott et al., 1962; Knudsen and Konishi, 1979; Gupta et al., 2021), which have been extensively mapped in various vertebrates, including teleost fishes (Popper and Fay, 1993; Fay and Popper, 2000; Gerhardt, 2015; Oliver et al., 2018).

Despite lacking structures directly comparable to the cochleae or the outer ear of mammals, teleost fish can perform complex auditory tasks such as frequency discrimination, locating sound sources, or analytic and synthetic listening (Schuijf et al., 1977; Fay, 1991; Cervi et al., 2012). In teleosts, sound is perceived through the inner ear maculae organs, with the sacculus being the most prominently studied one (Popper and Hokter, 1981; Higgs et al., 2003). Hair cells on the maculae organs are innervated by the eighth nerve that projects to the descending octaval nuclei (DON; Finger and Tong, 1984; Bass et al., 1994, 2000, 2001; Privat et al., 2019). Additional hindbrain auditory nuclei process auditory information, such as the anterior octaval nucleus (AON) and the secondary octaval population (SON). Ascending projections from the octaval nuclei reach the central nucleus of the torus semicircularis (TSc) in the midbrain via the lateral lemniscus (LL). TSc shares reciprocal connections to the central posterior nucleus (CP), which corresponds to the auditory thalamus in mammals (McCormick, 1999; Bass et al., 2000; Goodson and Bass, 2002; Mueller et al., 2004; Yáñez et al., 2024). From CP, auditory information reaches the dorsomedial division of the telencephalic pallium (Goodson and Bass, 2002; Northcutt, 2006; Casari Giassi et al., 2007; Yamamoto and Ito, 2008; Yáñez et al., 2024). Additional projections from TSc include the anterior tuberal nucleus (AT) in goldfish, Japanese carp, and channel catfish (Echteler, 1984; Striedter, 1991, Northcutt, 2006) and reciprocal connections in adult zebrafish and midshipman fish (Bass et al., 2000, 2001, Goodson and Bass, 2002; Yáñez et al., 2024). Interestingly AT receives input from the dorsomedial division of the telencephalic pallium, a structure known to receive and process auditory information (Goodson and Bass, 2002; Northcutt, 2006; Casari Giassi et al., 2007; Yamamoto and Ito, 2008).

Recent in vivo calcium imaging studies in larval zebrafish have largely confirmed the numerous anatomical and electrophysiological descriptions of teleost auditory pathways (McCormick, 1992, 1999; Schellart and Popper, 1992; Goodson and Bass, 2002). These studies revealed auditory-responsive neurons in the octaval nuclei, the TSc, the thalamus, the cerebellum, the tectum, and the pallium (Vanwalleghem et al., 2017; Privat et al., 2019; Constantin et al., 2020; Favre-Bulle et al., 2020; Poulsen et al., 2021). While these larval studies focused on key auditory areas, other potential auditory nuclei were unreported.

One such nucleus is AT, which is located within the ventrocaudal part of the hypothalamus. In the acoustically communicating midshipman fish, differences in the number of activated cells in AT have been found after acoustic stimulation with social stimuli (Petersen et al., 2013; Forlano et al., 2017; Mohr et al., 2018). The results suggested that AT is involved in processing acoustic social communication signals (Petersen et al., 2013; Forlano et al., 2017). Extracellular recordings also established AT's response to auditory information in goldfish in which auditory unimodal, bimodal (with vision or lateral line), and trimodal cells (vision and lateral line) were found (Kirsch et al., 2002). Whether AT in zebrafish is responsive to acoustic stimulation is currently unknown.

In the present study, we used tract tracing to investigate whether AT receives uniform or partially segregated inputs from TSc in adult zebrafish. To test the involvement of AT in auditory processing, we exposed zebrafish to broadband acoustic stimuli and measured neuronal activation indirectly with immunohistochemical visualization of phosphorylated ribosomal protein S6 (pS6). Although the pS6 physiological role in neurons is still debated, its phosphorylation is often used as a robust marker for neural activity in different vertebrates, including teleost fish (Knight et al., 2012; Biever et al., 2015; Meyuhas, 2015; Maruska et al., 2020; Tripp et al., 2020; Akinrinade et al., 2023). However, the time course of pS6 phosphorylation after neural activation is currently unreported in fishes. To establish a time series of pS6 phosphorylation in zebrafish, we collected animals at seven different time points after acoustic stimulation. We compared the number of pS6-immunoreactive (pS6-ir) cells to a baseline control group, which was not exposed to acoustic stimulation. Along with AT, we also measured the activation of two other brain regions: the central nucleus of the TSc and the nucleus isthmi (NI). Based on studies conducted on zebrafish larvae (Favre-Bulle et al., 2020), we anticipated that TSc would also respond to auditory stimulation in adult zebrafish. However, we did not expect NI, a midbrain visual processing center (Henriques et al., 2019) that we used as a control region, to react to auditory stimuli.

## Materials and Methods

### Subjects

Fifty-four adult zebrafish (*Danio rerio*; mean size, 30 mm; weight, 0.45 g) of both sexes were used for the study. Fish were housed in the animal facility at a constant temperature of 24.5–25°C in 200 L aquariums with a day/night cycle of 12 h. Four fish were used for the neural tracing study. One brain was lost during tissue processing. One of the animals was used for the negative controls of the primary antibodies by carrying out the staining protocols above but omitting the primary antibody. The remaining 48 were used for the brain activity measurements after acoustical stimulation. The experiment was carried out following the ethical guidelines of the local animal care committee's regulations.

### In vitro tracing

Animals were deeply anesthetized (0.05% MS222 in tank water) and subsequently decapitated. The skull was quickly removed to expose the brain in ice-cold Ringers’ solution for freshwater teleost fish (Kyriakatos et al., 2011). The upper part of the optic tectum of the left hemisphere was removed to expose the TS. Glass micropipettes (∼2–3 µm tips) containing 10% Neurobiotin (Vector Laboratories, SP-1120) in deionized water solution with 0.5 M potassium acetate (KCOOH) were positioned into the central division of the TS (TSc) using a micromanipulator (Narishige, model M-3333). A pulse generator (Grass Instruments, model SD9D) connected to a stimulus isolator (World Precision Instruments, model A365R) was used for the iontophoretic application of neurobiotin into TSc. Current pulses (10 µA with a stimulus duration of 200 ms at a frequency of ∼2 Hz) were continuously applied for 10 min.

After iontophoresis, brains were transferred for ∼7–8 h at 4°C in continuously carbonated Ringers’ solution to allow the neurobiotin to travel. Brains were then fixated overnight in 4% paraformaldehyde (PFA) in phosphate buffer solution (PB) at 4°C. One brain was cryoprotected in 30% sucrose in 0.1 M PB overnight at 4°C before being sectioned in the coronal plane in two consecutive series of 25 µm using a cryostat (Leica, model CM3000-1-). The other three brains were embedded in 4% agar, sectioned in the coronal plane at 75 µm on a vibratome (Campden Instruments, model 7000SMZ -0268), and directly mounted onto gelatin-coated slides. Mounted brain slides were washed in phosphate buffer saline solution (PBS; 0.1 M with 0.8% NaCl) and incubated for 2 h in a 1:500 streptavidin 647 (Invitrogen, S21374) in 0.03% PBS–Triton solution. Slides were then coverslipped with mounting medium (ROTH, ROTI Mount FluoCare with DAPI), and nail polish was applied on the slide borders to prevent the mounting medium from drying. Photographs were acquired with an epifluorescence microscope (Carl Zeiss, imager.M2; camera, Axiocam 305 mono) or on a confocal microscope (Leica Stellaris). Confocal images were composed from *z*-stacks acquired with a 20× objective and merged (maximum projection, maximum intensity mode) in ImageJ. Contrast and brightness were adjusted for the entire images.

### Experimental setup for acoustical stimulation

**Figure 1.**
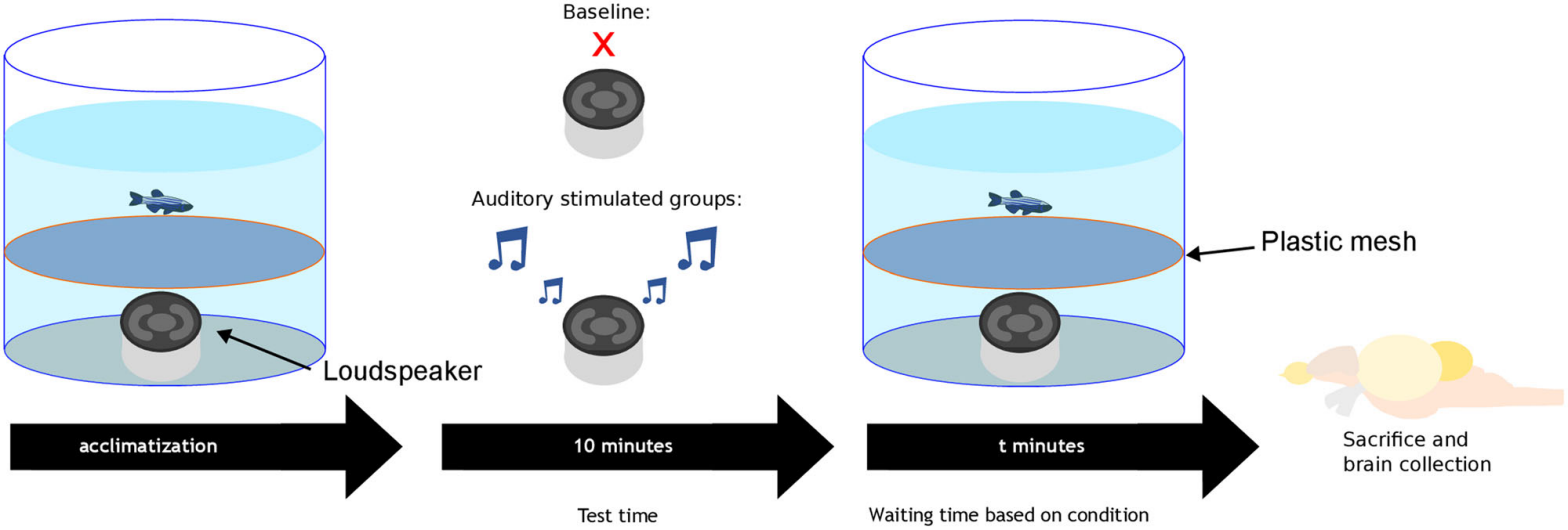
Experimental setup and procedure. Before auditory stimulation, fish were allowed to acclimatize to the enclosure overnight (left) before auditory stimulation began (middle). After auditory stimulation, fish were sacrificed at eight different time points (*t* = 0, 10, 70, 100, 130, 160, 190, and 250 min). The 0 minute time group served as a baseline control without auditory stimulation.

**Figure 2.**
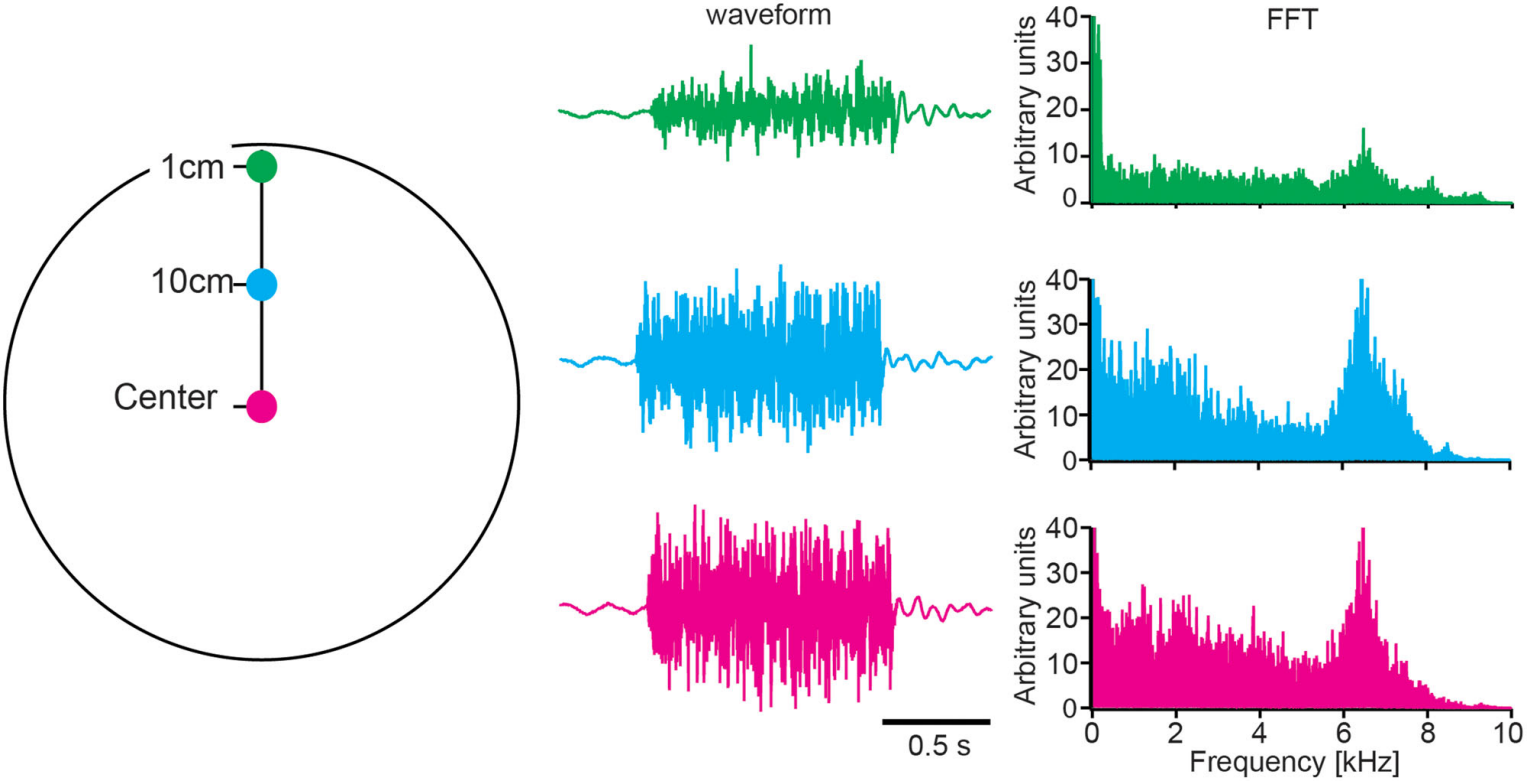
Illustration of the location within the experimental tank where the broad frequency range stimulus was measured (on the right). In the center of the figure, the corresponding waveforms of the auditory stimulation are displayed, with measurements taken at various distances from the tank's border: center (depicted in magenta), 10 cm from the border (depicted in cyan), and 1 cm from the border (depicted in green). Additionally, the fast Fourier transform (FFT) of the recorded stimuli in these different positions is presented on the right.

Fish were transferred from the animal facility to an environmentally controlled experimental room and placed in a cylindrical aquarium (31 × 34 cm; 28 L; Fig. 1). An underwater speaker (UW30, Lubell Labs) was positioned at the bottom of the aquarium with an aquarium pump and a heater. The speaker was stabilized by the aquarium substrate (small gravel). The speaker was driven by an amplifier (NAD Electronics, 214 stereo power amplifier) connected to a laptop computer from which audio files were played. Zebrafish possess accessory hearing structures that expand their hearing range (Higgs et al., 2003; Grande and Young, 2004). As their hearing covers frequencies from 100 to 4,000 Hz (Higgs et al., 2002; Cervi et al., 2012), we used broadband sounds to stimulate the auditory system. Audio files consisted of a series of frozen white noise lasting ∼0.1 s with 0.9 s pause looped for 10 min. Due to the nonlinearity of the speakers, the signal resulted in a broadband sound with a broad frequency range (Fig. 2). The resulting sound was measured with a hydrophone (Aquarian Scientific, model AS-1) at five positions in the tank (center, 5 cm, and 10 cm from the center for horizontal and longitudinal axis; see Fig. 2 for the schematic representation of the measured stimulus in one of the two axes). Hydrophone signals were amplified (Aquarian Scientific, model PA-4) and stored at 40 kHz resolution as wave files. To analyze the spectral pattern of the recorded sound, we performed Fourier analysis in Igor Pro 7 (WaveMetrics).

A thin plastic mesh was placed 10 cm above the loudspeaker, which prevented the fish from accessing the sound source. Fish were allowed to swim freely in the upper compartment. Individual fish were placed into the arena the evening before the experiment to allow for habituation to the experimental setup. The evening before the auditory stimulation test, the pump and the heater in the tank were turned off to prevent acoustic and lateral line stimulation. Fish were tested the following morning. The aquarium tank temperature was 23°C. Ambient lights were on in the experimental room during the duration of the experiment, but no additional light source illuminated the experimental aquarium.

### Acoustic stimulation

Fish were divided into eight groups, with six subjects per group. Each group refers to different time points in which experimental fish remained in the tank after the onset of sound exposure: 0 min (baseline group, no auditory stimulation), 10, 70, 100, 130, 190 and 250 min. Individual fish were exposed for 10 min to the broadband auditory stimulus. The amplitude of the sound did not exceed the sound intensity in the home tanks induced by circulating pumps and aeration (root mean square, 0.2523–0.2571). Once the allocated time in the tank was reached, the fish were removed from the aquarium, deeply anesthetized with 0.05% tricaine methane sulfonate (MS-222) dissolved in tank water, and euthanized by cervical transection. The fish brains were rapidly exposed and immersion fixed for 1–2 h in cold 4% PFA in 0.1 M PBS. The brains were removed from the skulls and stored in 0.1 M PBS for 1–7 d at 4°C.

### Immunohistochemistry

The brains were cryoprotected in 30% sucrose in 0.1 M PBS overnight at 4°C. They were then sectioned in two series at 25 µm on a cryostat and mounted directly onto gelatin-coated slides. The brains were cut in a coronal plane, ensuring the brain sections had a similar orientation to those in the zebrafish brain atlas (Wullimann et al., 1996). Slides were dried under a fume hood overnight at room temperature and stored at −20°C until processing. One series was used for immunohistochemistry to evaluate pS6 phosphorylation, while the other served as a backup.

Dried slides were washed in PBS for 3 × 5 min. Unspecific binding sites were blocked with 2% normal donkey serum (0171-000-121, Jackson ImmunoResearch Laboratories, INC.) in PBS containing Triton (0.3%) for 1 h at room temperature and then washed in PBS for 3 × 5 min. Sections were incubated with anti-pS6 antibody (1:500; Cell Signaling pS6 ribosomal protein S235/236 antibody 2211, made in rabbit with species cross-reactivity in zebrafish; Cell Signaling website; RRID, P62753) and anti-choline acetyltransferase (ChAT) antibody (1:500; ChAT, AB144P; Merck Millipore; RRID, AB_2079751, made in goat) overnight at 4°C in 2% normal donkey in PBS–Triton solution. This ChAT antibody was previously used for Western blotting in zebrafish (Clemente et al., 2004; Beckers et al., 2023), and in the current study, it served as an anatomical marker of the NI (Mueller et al., 2004; Henriques et al., 2019). After three washes with PBS, slides were incubated for 60 min at room temperature with secondary antibodies in PBS–Triton with 2% normal donkey blocking solution. Alexa Fluor 546-conjugated anti-rabbit (1:500; Invitrogen, A10040, made in donkey) was used to label pS6 and Alexa Fluor 488-conjugated anti-goat (1:500; Invitrogen, A11055, made in donkey) to label ChAT. Slides were quickly rinsed in double distilled water and coverslipped with mounting medium (ROTH, ROTI Mount FluoCare with DAPI). The slides were sealed with nail polish. We tested for nonspecific antibody binding to the tissue for all primary antibodies by carrying out the staining protocols above but omitting the primary antibodies. All tests revealed no staining.

### pS6 quantification

**Figure 3.**
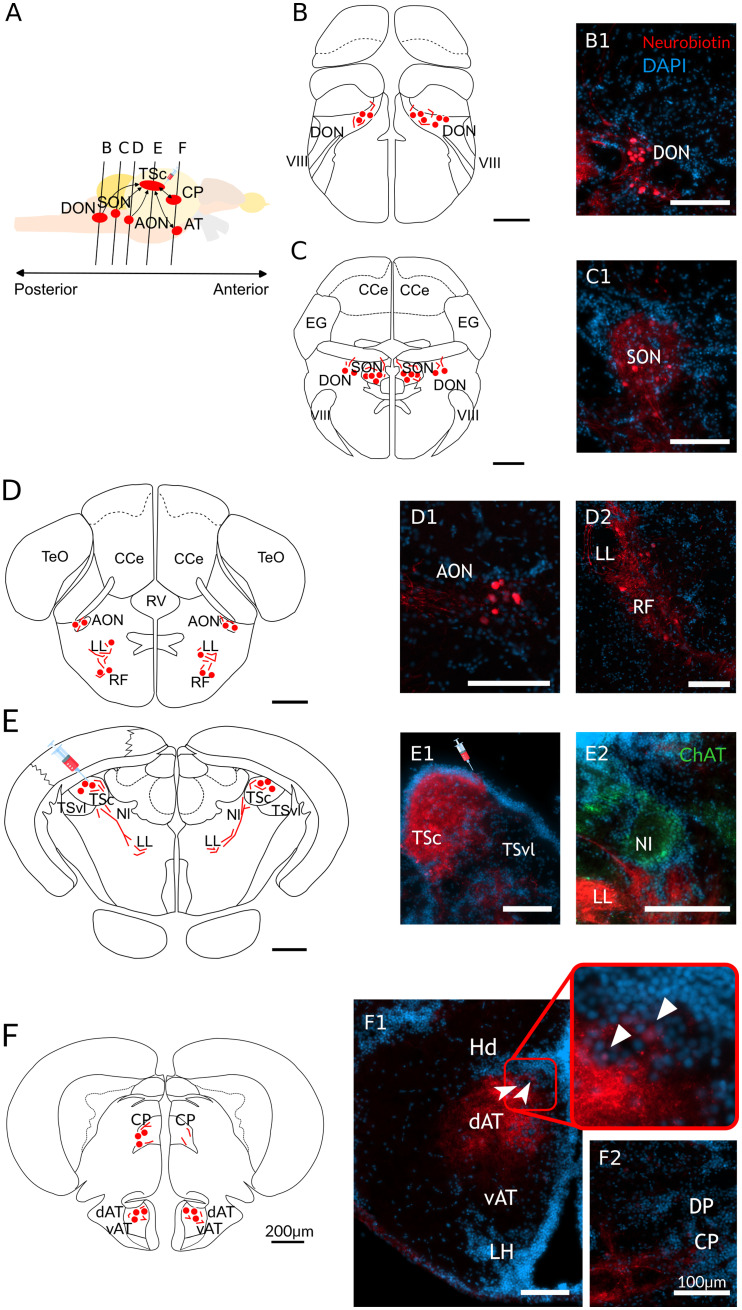
Summary of tracing in the central division of the TSc. ***A***, Schematic drawing of the zebrafish brain from the sagittal view highlights the ascending auditory regions and the coronal plane locations (***B–F***) along the anterior–posterior axis. ***B–F***, Schematic drawings of the coronal sections showing the distribution of labeling (red circles, cell bodies; red lines, fibers) and the corresponding photographs: Neurobiotin-labeled cells and fibers in DON (***B*1**), in SON (***C*1**), in AON (***D*1**); in the LL and RF (***D*2**) and the injection site in the TSc (***E*1**). Please note that the fibers visible in the TSvl do not terminate in this region and are projections from TSc to other regions; ***E*2**, The location of NI, delineated based on ChAT staining in which no Neurobiotin was found. ***F***, Labeling in the AT is limited to dAT and is absent in the vAT region. Inset in ***F*1** shows a magnification in the dAT in which labeled cell bodies were present, as indicated by the white arrows. ***F*2**, Labeling in the CP. Scale bars represent 200 µm in the schematic drawings and 100 µm in the staining sections. DON, dorsal octaval nucleus; SON, secondary octaval nucleus; AON, anterior octaval nucleus; LL, lateral lemniscus; RF, reticular formation; CP, central posterior thalamic nucleus; AT, anterior tuberal nucleus; TSc, the central nucleus of the torus semicircularis; NI, nucleus isthmi; CP, central posterior thalamic nucleus; CCe, corpus cerebelli; EG, eminentia granularis; VIII, octaval nerve; TeO, tectum opticum; TSvl, ventrolateral nucleus of torus semicircularis; RV, rhombencephalic ventricle; vAT, ventral part of the anterior tuberal nucleus; DP, dorsal posterior thalamic nucleus; LH, lateral hypothalamic nucleus; DP, dorsal zone of periventricular nucleus.

pS6-labeled brain sections were examined with epifluorescence microscopy (Thorlabs CSB2200 or Zeiss Imager M2), and photographs were taken with a digital camera (Thorlabs 1501M or Axiocam 305 mono). Counting of pS6-ir cells was performed with Fiji (ImageJ 1.53f51) blind to the experimental conditions. Brain areas were identified based on the DAPI staining using the reference atlas for adult zebrafish (Wullimann et al., 1996). In each brain area, the counting areas were positioned over the spots with the highest number of pS6-ir cells (Zangenehpour and Chaudhuri, 2002; Heimovics and Riters, 2006; Knight et al., 2012; Corrales Parada et al., 2021; Morandi-Raikova et al., 2021; Polzin et al., 2021; Adiletta et al., 2022; Protti-Sánchez et al., 2022; Asogwa et al., 2023). To quantify pS6-ir cells in AT, four sections of both hemispheres were selected from cross sections 136–153 according to the zebrafish atlas (Wullimann et al., 1996). AT in each section was subdivided into a dorsal (dAT) and a ventral region (vAT). This division was based on the tracing experiments (see our results). We measured the time course of pS6 phosphorylation in dAT of all time groups. Activation of vAT was measured only in the group with the highest and lowest pS6 activation (100 min and baseline groups in auditory regions; see Results). A square counting area (100 × 100 μm) was placed over AT's dorsal and ventral regions in each section and hemisphere. Ten sections were used for quantification of pS6 in the central nucleus of TS, which is at the border with nucleus laterals valvulae and the dorsal part of the ventrolateral nucleus of the TS (TSvl). The counting area (150 × 150 μm) was placed on the most medial part of TSc (Fig. 3*E*,*E*1 for the anatomical localization of the brain region). Three sections containing NI were identified with the anti-ChAt as an anatomical marker (Mueller et al., 2004; Henriques et al., 2019). In all fish, only one section of each series contained the central part of NI. A square counting area (100 × 100 μm) was used to fit the size of NI.

Photographs were made of the counting areas in all regions. These images were transformed to 8 bits and despeckled in Fiji (version 1.53, NIH) to improve the visibility of activated cells during the counting procedure. pS6-positive cells were manually marked using the cell counter in Fiji (version 1.53, NIH). We measured the cell densities of each brain region obtained from different sections. These cell densities were averaged across sections and standardized to cells/mm^2^. Since there were no visually apparent differences between the hemispheres in any of the brain regions, the hemispheres were averaged. The resulting values were considered representative of the overall activity in each brain region for each individual and were employed for further statistical analysis.

### Statistical analysis

Since the residuals of the measured cell densities in some brain regions of interest were not normally distributed (Shapiro–Wilk test dAT, *w* = 0.924; *p* = 0.004; TSc, *w* = 0.976; *p* = 0.419; NI, *w* = 0.958; *p* = 0.080), we used nonparametric statistics. To compare the pS6 phosphorylation of the eight-time groups, we employed three Kruskal–Wallis tests, one for each brain area (dAT, TSc, and NI). For post hoc analysis, we used the Dunn’s test. For the analysis of region-specific activation, we used a nonparametric aligned rank transformation ANOVA with two factors (group and area). For the post hoc analysis, we performed Dunnett’s tests. All statistical analysis was carried out in RStudio R Core Team (2023), using the following packages: “rcompanion”; “FSA”; “tidyverse”; and “ARTool.” The plots were generated in GraphPad Software (Version 9.5.0).

## Results

### Connectivity between TSc and AT

**Figure 4.**
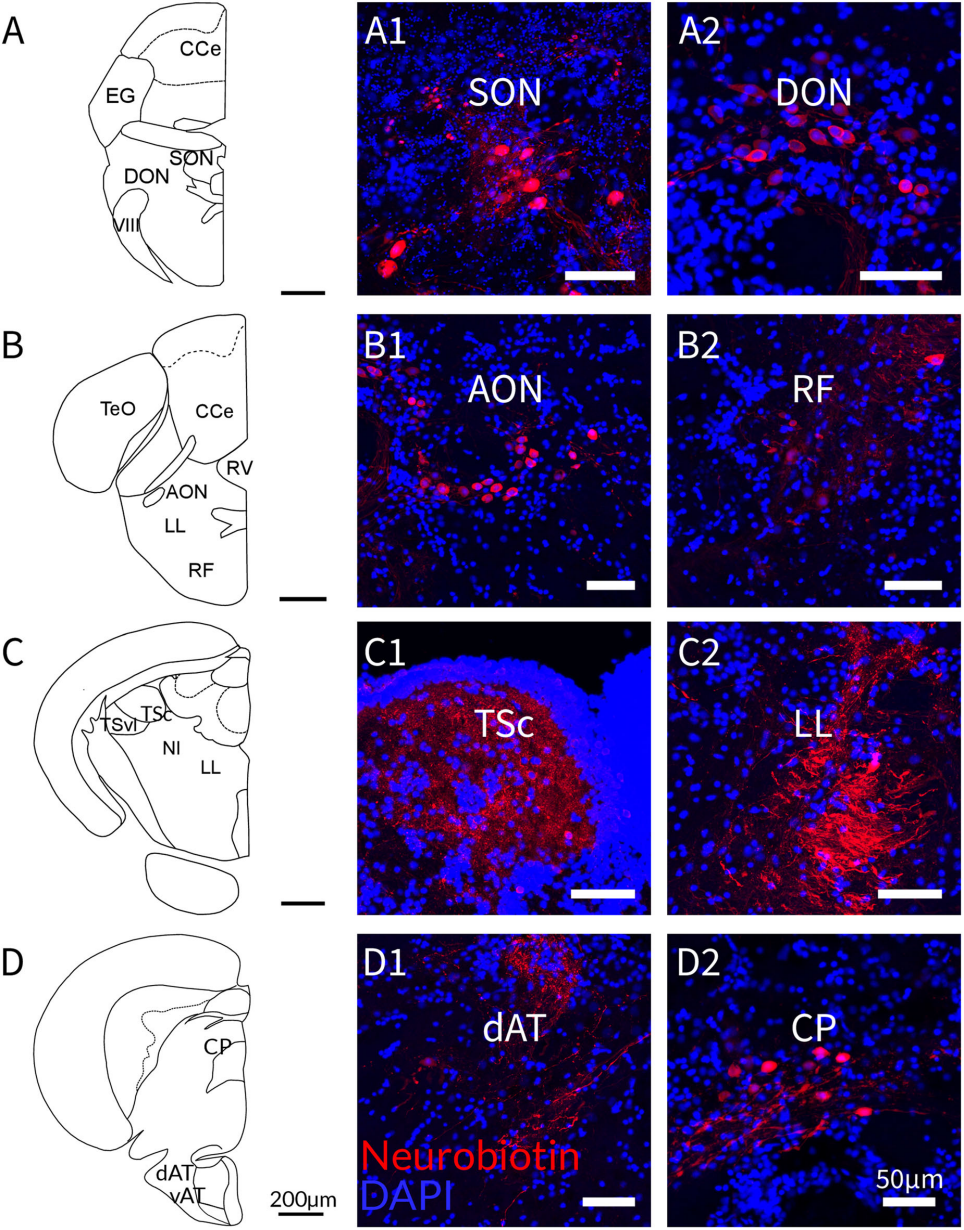
Maximum projections of confocal micrographs depicting the main toral connectivity. Schematic drawings of coronal brain sections and photographs of connected nuclei. Neurobiotin-labeling was found in the SON (***A*1**), the DON (***A*2**), the AON (***B*1**), the RF (***B*2**), fibers of the LL (***C*2**), the dAT (***D*1**), and CP (***D*2**). Further depicted is the injection site in the central nucleus of TSc (***C*1**) with labeled fibers and cells. CCe, corpus cerebelli; EG, eminentia granularis; VIII, octaval nerve; TeO, tectum opticum; TSvl, ventrolateral nucleus of torus semicircularis; RV, rhombencephalic ventricle; vAT, ventral part of the anterior tuberal nucleus; DP, dorsal posterior thalamic nucleus; LH, lateral hypothalamic nucleus; DP, dorsal zone of periventricular nucleus. Scale bars are 200 µm in the schematics and 50 µm in the confocal projections.

Our tracing experiments revealed a reciprocal connectivity between the zebrafish's AT and the central nucleus of the TSc. After Neurobiotin injections in the TSc (see Figs. 3*E*1, 4*C*1 for a detailed figure of the labeling in TSc), we found robust labeling with putative terminals, cells, and fibers in AT (Fig. 3*F*,*F*1; see Fig. 4*D*1 for a detailed figure of the labeling in dAT). This labeling was present both ipsilaterally and contralateral to the injection site and delineated specifically the dAT. The labeling was prominent along the rostrocaudal extent of dAT, implying strong connectivity of this AT subregion with the TSc.

In addition, and in line with previous results from adult zebrafish (Yáñez et al., 2024), we also found staining in other auditory-related regions: we found fibers, putative terminals, and some somata in the contralateral TSc and bilateral labeling in the CP. The labeling in CP (Fig. 3*F*,*F*2; see Fig. 4*D*2 for a detailed figure of the labeling in CP) was predominantly ipsilateral, but fibers and putative terminals were also found on the contralateral side. In the hindbrain, fibers and labeled somata were found in the DON (Fig. 3*B,B*1; see Fig. 4*A*2 for a detailed figure of the labeling in DON). Labeling was bilateral, with an apparent larger distribution on the contralateral side. In SON (Fig. 3*C,C*1; see Fig. 4*A*1 for a detailed figure of the labeling in SON), another octaval nucleus, cells, and fibers were abundant bilaterally. Labeled somata and fibers were also found in the AON (Fig. 3*D*,*D*1; see Fig. 4*B*1 for a detailed figure of the labeling in AON). Other labeled regions included fibers in the LL and fibers and somata in the reticular formation (RF; Fig. 3*D*,*D*2; see Fig. 4*C*2,*B*2 for a detailed figure of the labeling in LL and RF). Other reciprocal projections included the preoptic area and the preglomerular complex.

### Time course of pS6 phosphorylation after acoustical stimulation

**Figure 5.**
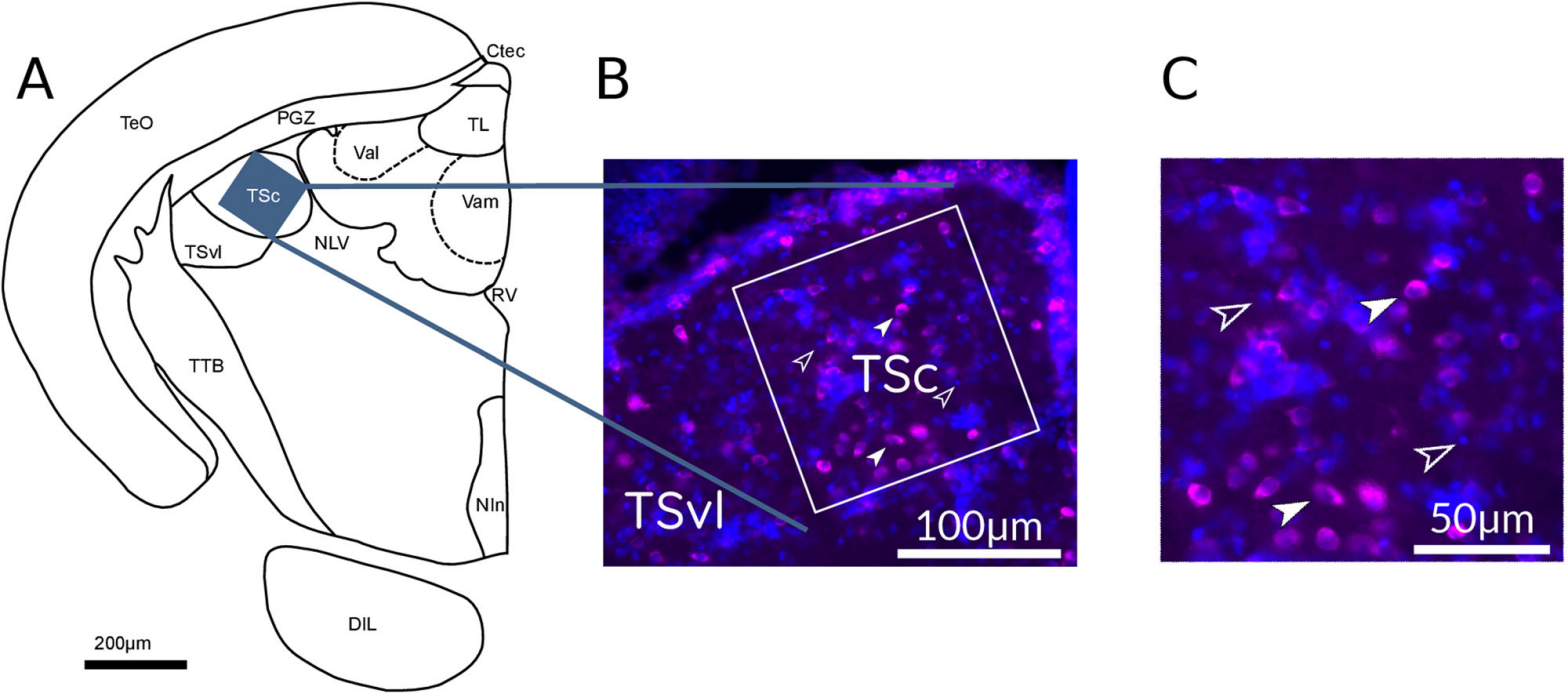
***A***, Schematic drawing of a coronal section with a typical placement of a cell counting zone (blue rectangle) within the central nucleus of the TSc. ***B***, Example photograph of a pS6 staining and the positioned counting zone within the TSc. ***C***, Zoom to the cell counting zone within TSc. pS6-ir cells are stained in magenta (full arrowheads) and are easily distinguishable from the nonactivated cells (empty arrowheads). Cell nuclei are stained with DAPI (blue). DIL, diffuse nucleus of the anterior lobe; Nin, nucleus intrapeduncularis; TTB, tractus tectobulbaris; NLV, nucleus lateralis valvulae; TSvl, ventrolateral nucleus of torus semicircularis (TSvl); TL, torus longitudinalis; TeO, tectum opticum; PGZ, periventricular gray zone of optic tectum; RV, rhombencephalic ventricle; Ctec, commissura tecti; Val, lateral division of valvula cerebelli; Vam, medial division of valvula cerebelli.

**Figure 6.**
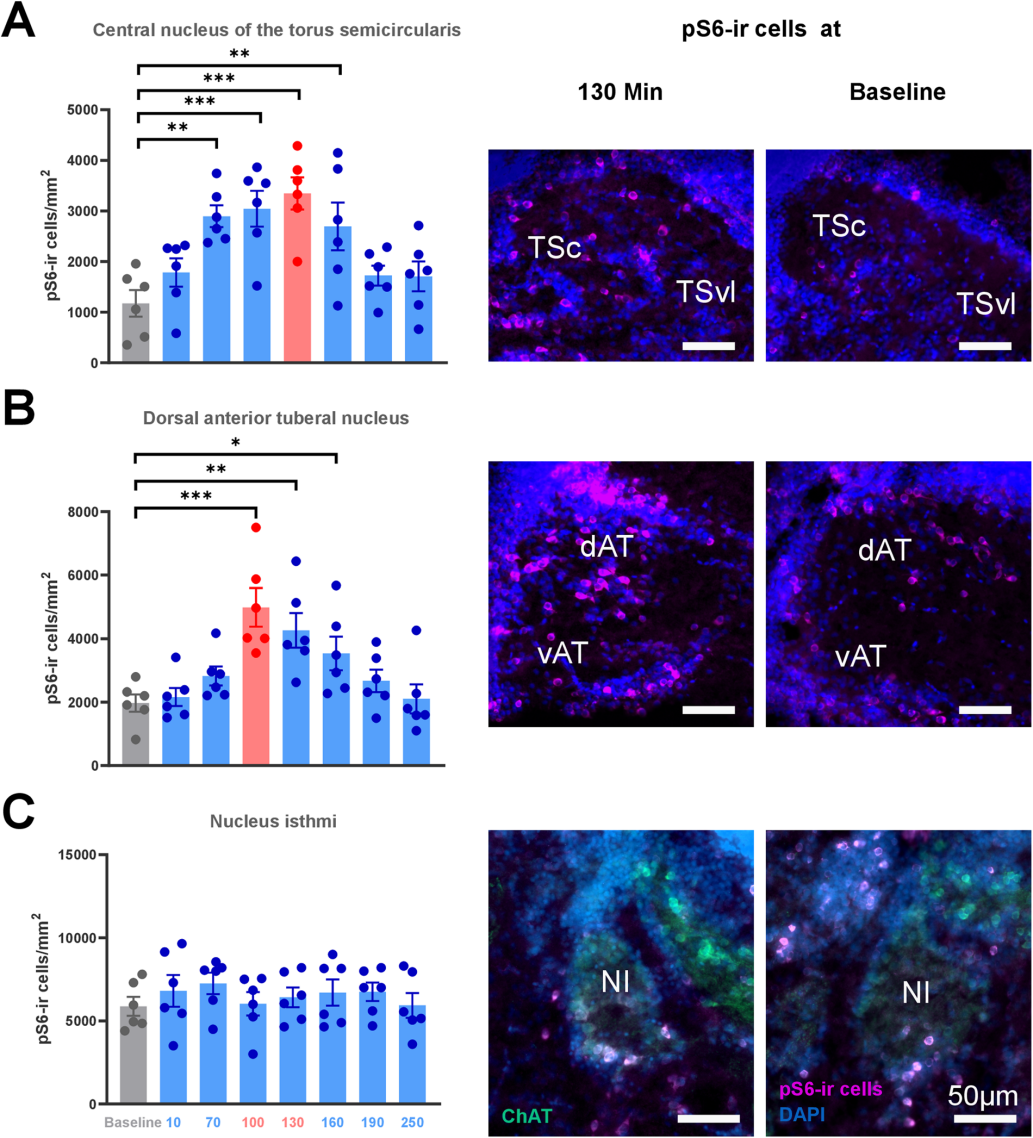
The time-dependent pattern of up- and downregulation of pS6 phosphorylation across the three regions of interest. ***A***, Central nucleus of the TSc. ***B***, Dorsal portion of the anterior tuberal nucleus (dAT). ***C***, NI. Bar plots show mean ± SEM with dots representing the individual values. Time groups in which the peak level was reached are in red. The unstimulated baseline is in gray and the other bars in blue. Asterisks mark significant differences compared with the baseline (*p < 0.05; **p < 0.01). The right side of the figure depicts representative example photos of pS6-ir cells (magenta) of the 130 min and baseline time groups in the three brain regions with DAPI (blue) and the ChAT (green, used only to delineate NI).

We determined pS6 phosphorylation at different time points after auditory stimulation in TSc, Dat, and NI. All 48 brains (six in each time group) were successfully stained and provided easily detectable pS6-labeled cells (Figs. 5, 6). The measured pS6-ir cell densities are summarized in Table 1. In the TSc, we found a time-dependent pattern of up- and downregulation of pS6 phosphorylation (Fig. 6*A*) with significant differences between the time groups (Kruskal–Wallis *H*_(7)_ = 25.998; *p* < 0.001). In the post hoc comparisons (Table 2), a significant difference from the baseline was first observed 70 min after the stimulation onset (baseline, 1,174.1 ± 263 cells/mm^2^; 70 min, 2,895.9 ± 215 cells/mm^2^; *p* = 0.002). The highest number of pS6-ir cells in the TSc was observed at 130 min (3,043.7 ± 355 cells/mm^2^; *p* < 0.001), while the difference compared with baseline levels disappeared for the first time at 190 min (1,723.5 ± 196 cells/mm^2^; *p* = 0.409).

**Table 1.** pS6-ir cell densities across the time groups in the TSc, dAT, and NI (mean ± SEM, rounded numbers)

Time groups	TSc	dAT	NI
0	1,174.1 ± 263	1,970.8 ± 272	5,875 ± 572
10	1,785.1 ± 280	2,162.5 ± 285	6,808.3 ± 959
70	2,895.9 ± 215	2,825 ± 299	7,258.3 ± 642
100	3,043.7 ± 355	4,989.6 ± 606	6,025 ± 706
130	3,346.8 ± 319	4,264.6 ± 544	6,416.7 ± 593
160	2,694.9 ± 472	3,536.8 ± 531	6,708.3 ± 788
190	1,723.5 ± 196	2,674.3 ± 353	6,750 ± 557
250	1,706.8 ± 296	2,104.2 ± 459	5,933.3 ± 745

**Table 2.** Results of the post hoc Dunn’s tests for TSc and dAT with significant *p* values in bold

Comparison between time groups	TSc	dAT
0 vs 10	*Z* = 1.093; *p* = 0.274	*Z* = 0.124; *p* = 0.902
0 vs 70	*Z* = 3.134; ***p* = 0.002**	*Z* = 1.454; *p* = 0.146
0 vs 100	*Z* = −3.175; *p* = 0.001*	*Z* = −3.464; *p* = 0.001*
0 vs 130	*Z* = 3.629; *p* < 0.001*	*Z* = 2.928; ***p* = 0.003**
0 vs 160	*Z* = 2.577; ***p* = 0.010**	*Z* = 2.217; ***p* = 0.027**
0 vs 190	*Z* = 0.825; *p* = 0.409	*Z* = 1.000; *p* = 0.317
0 vs 250	*Z* = 0.907; *p* = 0.364	*Z* = 0.031; *p* = 0.975
10 vs 70	*Z* = −2.041; ***p* = 0.041**	*Z* = −1.330; *p* = 0.184
10 vs 100	*Z* = −2.083; ***p* = 0.037**	*Z* = −3.341; *p* = 0.001*
10 vs 130	*Z* = −2.536; ***p* = 0.011**	*Z* = −2.804; ***p* = 0.005**
10 vs 160	*Z* = −1.485; *p* = 0.138	*Z* = −2.093; ***p* = 0.036**
10 vs 190	*Z* = 0.268; *p* = 0.789	*Z* = −0.876; *p* = 0.381
10 vs 250	*Z* = 0.186; *p* = 0.853	*Z* = 0.093; *p* = 0.926
70 vs 100	*Z* = −0.041; *p* = 0.967	*Z* = −2.011; ***p* = 0.044**
70 vs 130	*Z* = 0.495; *p* = 0.621	*Z* = 1.474; *p* = 0.140
70 vs 160	*Z* = −0.557; *p* = 0.578	*Z* = 0.763; *p* = 0.445
70 vs 190	*Z* = −2.309; ***p* = 0.021**	*Z* = −0.454; *p* = 0.650
70 vs 250	*Z* = −2.227; ***p* = 0.026**	*Z* = −1.423; *p* = 0.155
100 vs 130	*Z* = 0.454; *p* = 0.650	*Z* = −0.536; *p* = 0.591
100 vs 160	*Z* = −0.598; *p* = 0.550	*Z* = −1.248; *p* = 0.212
100 vs 190	*Z* = −2.351; ***p* = 0.019**	*Z* = −2.464; ***p* = 0.014**
100 vs 250	*Z* = −2.268; ***p* = 0.023**	*Z* = −3.433; *p* = 0.001*
130 vs 160	*Z* = 1.052; *p* = 0.293	*Z* = 0.711; *p* = 0.477
130 vs 190	*Z* = 2.804; ***p* = 0.005**	*Z* = 1.928; *p* = 0.054
130 vs 250	*Z* = 2.722; ***p* = 0.006**	*Z* = 2.897; ***p* = 0.004**
160 vs 190	*Z* = 1.753; *p* = 0.080	*Z* = 1.217; *p* = 0.224
160 vs 250	*Z* = 1.670; *p* = 0.095	*Z* = 2.186; ***p* = 0.029**
190 vs 250	*Z* = −0.082; *p* = 0.934	*Z* = 0.969; *p* = 0.332

* Significant also after a Bonferroni’s correction for multiple comparisons.

In dAT, we found a similar pattern of up- and downregulation of pS6 phosphorylation (Kruskal–Wallis: *H*_(7)_ = 25.779; *p* < 0.001; Fig. 6*B*). The post hoc analysis (Table 2) revealed a first significant difference in comparison with the baseline at 100 min (baseline, 1,970.8 ± 272 cells/mm^2^; 100 min, 4,989.6 ± 606 cells/mm^2^; *p* = 0.001) which was also the peak activation. The difference to the baseline disappeared for the first time at 190 min (2,674.3 ± 353 cells/mm^2^; *p* = 0.317). In NI, pS6 labeling showed no significant differences across the different time groups (Fig. 6*C*; Kruskal–Wallis, *H*_(7)_ = 4.123; *p* = 0.766).

### The response to auditory stimulation in AT is specific to its dorsal portion

**Figure 7.**
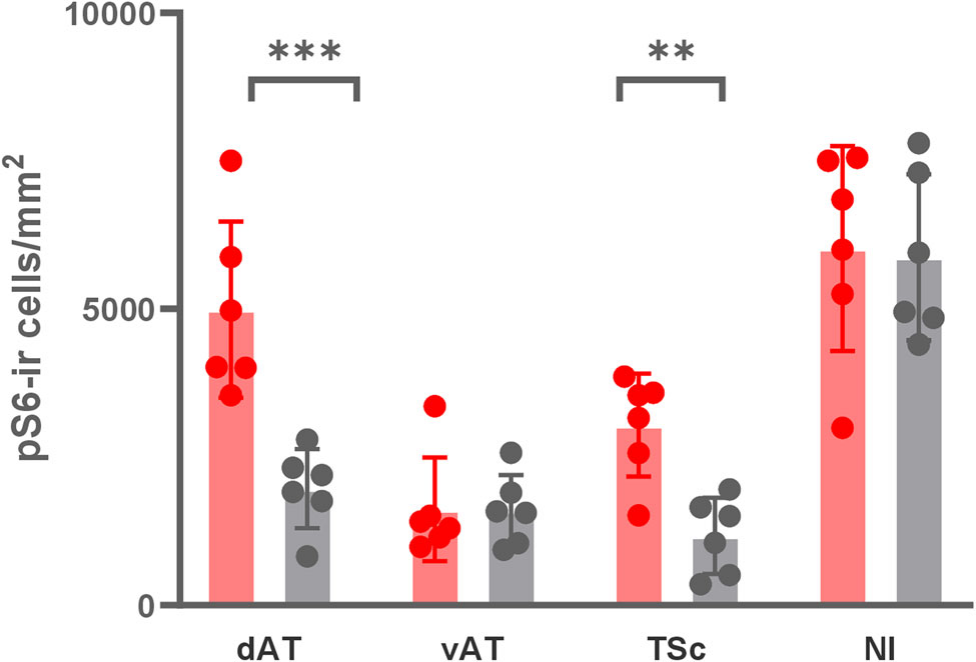
pS6-ir cell densities per mm^2^ in the 100 min (red) and baseline groups (gray) across regions of interest: dAT and vAT, central nucleus of the TSc and NI. Bar plots show mean ± SEM, with dots representing the individual values (**p < 0.01; ***p <0.001).

To further investigate if the responses to auditory stimulation were specific to only the dAT, we focused on the time group at 100 min, which had the highest number of pS6-ir cells in this brain region. We thus quantified vAT only in this time group and the baseline. We then performed an aligned rank transformation ANOVA with the factors groups (two levels, 100 and 0 min) and brain regions (four levels, dAT, vAT, TSc, NI). The analysis revealed a significant main effect of groups (*F*_(1,66)_ = 17.510; *p* < 0.001), brain regions (*F*_(1,66)_ = 32.985; *p* < 0.001), and a significant interaction of the two factors (*F*_(1,66)_ = 5.426; *p* = 0.003). This indicates that pS6-ir cell densities differed between the two groups in a brain region-specific way. The post hoc analysis revealed that the activation of vAT at 100 min (1,620.8 ± 357 cells/mm^2^) was not different from the baseline (1,602.1 ± 244 cells/mm^2^; *t*_(40)_ = 0.365; *p* = 1.000). The differences were significant only in dorsal AT (*t*_(40)_ = −4.602; *p* = 0.001) and TSc (*t*_(40)_ = −3.974; *p* < 0.007) but not in NI (*t*_(40)_ = −0.061; *p* = 1.000; Fig. 7).

## Discussion

The present study revealed a functional and anatomical subdivision of zebrafish's AT. Our tracing experiments showed that only dAT is strongly reciprocally connected to the central nucleus of the TSc, a central auditory nucleus. In line with the tracing experiment, the indirect neural activity measurements revealed that the dAT, but not the vAT, responded to auditory stimulation. As expected from these tracings experiments, the density of pS6 phosphorylated cells was higher in dAT and TSc of zebrafish collected 100 min after auditory stimulation than in the nonstimulated baseline. Such differences between the groups were absent in vAT and NI (a visual area that does not process auditory information), indicating that the response to auditory stimulation was brain region-specific. Furthermore, we have determined the timeline of the increase and decrease of pS6 phosphorylation after acoustical stimulation. The peak activation was observed at 100 min in dAT and 130 min in TSc. In both brain regions, the activity returned to the baseline at 190 min after stimulation.

Our results suggest that AT is an auditory processing nucleus. The strong connection between TSc and AT is in line with previous reports in midshipman fish, zebrafish, goldfish, and Japanese carp, in which putative terminals were found in AT after tracing the connectivity of TSc (Echteler, 1984; Kozloski and Crawford, 1998; Bass et al., 2000, 2001; Northcutt, 2006; Yáñez et al., 2024). In our experiments, injections of Neurobiotin, an anterograde and retrograde tracer, in TSc exhibited its reciprocal connections to various key auditory processing regions, including the DON, SON, AON, and CP, making it a central hub in this network. Moreover, we found that TSc is strongly connected to the dorsal portion of AT but not to the ventral parts of AT. In line with the connectivity pattern, both the TSc and the dorsal AT responded to acoustic stimulation, but not the ventral AT. Thus, our study revealed a functional and anatomical subdivision of AT, which was not reported in previous studies in teleost. This region may thus respond preferentially to other sensory modalities, such as hydrodynamic and visual processing also present in the AT (Kirsch et al., 2002). To test this hypothesis, further investigations are necessary.

Our results align with electrophysiological studies in goldfish, which show that neurons in AT respond to auditory stimuli (Kirsch et al., 2002). Since we used acoustical stimulation, the majority of pS6-ir cells in our study indicate auditory responses. However, we cannot fully exclude that other modalities could have also influenced the activation. In goldfish, AT receives input from the dorsomedial pallium, which is a multisensory area (Northcutt, 2006), while this is not the case in zebrafish (Yáñez et al., 2022). Consequently, unimodal, bimodal, and trimodal units have been found in goldfish AT after sensory stimulation (visual, acoustic, or hydrodynamic), indicating that AT responds to different sensory modalities (Kirsch et al., 2002). In our study, all fish were exposed to the same visual environment, reducing the influence of visual information. However, the acoustical stimulation that was used could have caused a change in the fish's behavior, which we did not record. Since the sensory input that an animal receives is directly linked to its motor activity, this potential change in behavior could have caused an increase in visual (optic flow) and lateral line stimulation. However, a major impact of visual information is unlikely in our study since the pS6 activity in the visual area NI did not show any differences between the groups. Thus, visual information alone cannot explain the differences in activation of dAT and TSc we found between the groups. We also believe that any potential lateral line activation did not play a significant role. Notably, the lateral line primarily projects to the TSvl rather than TSc (Bleckmann, 2008; Wullimann and Grothe, 2014). Since the TSc is an auditory nucleus, the similar activation of dAT at similar time points as TSc and lack of activation in other regions suggest that the acoustic stimulus was the driver of pS6 phosphorylation in these regions. Future studies could focus on injecting neurobiotin into TSvl and investigate if also lateral line information is directly transmitted to dAT.

Our current study measured neural activation using the phosphorylation of the pS6. This method enabled us to assess the activation of the AT (and its functional division), which was not reported through calcium imaging in zebrafish larvae (Vanwalleghem et al., 2017; Privat et al., 2019; Constantin et al., 2020; Favre-Bulle et al., 2020; Poulsen et al., 2021). Our time course measurements revealed a peak of pS6 phosphorylation at 100 min in TSc and 130 min in AT after auditory stimulation. This suggests that there might be a variation in the time course of pS6 phosphorylation between the two brain regions. Nevertheless, to validate this hypothesis, the study would need a larger sample size. With the present data, a more likely interpretation is that the different time points for the two brain areas were due to statistical variability. In both brain regions, there was a significant difference at time points 100 and 130 min when compared with the baseline. However, there was no significant difference between time points 100 and 130 min (Table 2). This suggests that the difference in peak response between the two brain areas was not substantial. Thus, based on our study, the time point between 100 and 130 min (120 min) after stimulus presentation would be the optimal time for detection of high levels of pS6 phosphorylated cells. This result is in line with previous studies showing colocalization of pS6 and c-Fos at 2 h after behavioral and pharmacological stimulations in mice (Knight et al., 2012; Petersen et al., 2013). Our finding of a similar time course thus contributes to the growing evidence that pS6 phosphorylation is linked to the expression of immediate early genes. S6 is a component of the ribosome's structure that gets phosphorylated downstream of signaling pathways that also govern the transcription of neural activity-dependent immediate early genes like *c-fos* (Flavell and Greenberg, 2008; Meyuhas, 2015). The use of immediate early genes products as markers of neural activity is a common procedure (Herrera and Robertson, 1996; Kovács, 1998). *c-fos* expression is currently one of the most used indirect markers of neural activity because its products mRNA and the c-Fos protein rapidly accumulate in activated neurons (Herrera and Robertson, 1996; Kovács, 1998). The peak levels of the *c-fos* mRNA are reached at ∼20–30 min after neural activation, while the peak level of the protein is between 1 and 2 h after stimulation (Zangenehpour and Chaudhuri, 2002). The time course of a similarly popular immediate early gene (*egr-1*) expression has also been investigated in fish, showing similar results (Burmeister and Fernald, 2005). Our time course of pS6 phosphorylation complements these studies. For the design of activity mapping studies, it is crucial to determine not only the peak responses but also the return to the baseline level. For example, the transportation of animals to the experimental setup may lead to neural activation and consequently an increase in phosphorylation of pS6, which should be allowed to return to its baseline levels before conducting experiments. According to our findings, the minimum time for habituating the animals before any stimulation is 190 min. This is because the levels of pS6 in our study were not significantly different from the baseline levels at this point. This information will be valuable for the design and standardization of future pS6 experiments.

## Conclusion

Our findings show a prominent connection of the acoustic system to dAT and activation of dAT after acoustic stimulation, in line with the diverse response profiles to acoustic stimulation reported for AT in goldfish (Kirsch et al., 2002). Further anatomical and physiological studies are needed to investigate if a similar organization of lateral line and visual information are present in AT. Such studies should include tract tracing and physiological responses to the lateral line and/or visual stimulation to better understand how different sensory modalities are processed in AT. Colocalization studies of pS6 with other neural types’ specific markers would further contribute to unveiling the neural circuits involved.

## Synthesis

Reviewing Editor: Luke Remage-Healey, University of Massachusetts Amherst Decisions are customarily a result of the Reviewing Editor and the peer reviewers coming together and discussing their recommendations until a consensus is reached. When revisions are invited, a fact-based synthesis statement explaining their decision and outlining what is needed to prepare a revision will be listed below. The following reviewer(s) agreed to reveal their identity: Mario Wullimann.

The manuscript was evaluated by two expert reviewers. They are in good support of the study and lay out a set of concerns to be addressed. Chiefly among these is the shared concern that an improved, objective, and standardized counting method would greatly enhance the reliability of the pS6 expression counts. This amounts to a refocus on the entire brain region and standardized evaluation of expression by section number for each animal, which would address concerns about reliability estimates. It is likely that confocal images need to be re-acquired for this purpose.

Reviewer 1

The paper „The dorsal part of the anterior tuberal nucleus responds to auditory stimulation in zebrafish (Danio rerio)" describes the involvement of a hypothalamic nucleus, the dorsal division of the anterior tuberal nucleus, in auditory circuitry and function in the zebrafish brain. This represents progress in the understanding of this auditory subsystem which is particular to teleosts and absent in land-vertebratres. It has been desribed before in a selection of teleost species. However, the use of ribosomal protein S6 (pS6) phosphorylation in a nicely thought out experiment demonstrates here plausible physiological data to support such a function. The paper is well written, the figures explain the findings highly plausibly (but see below).

I have only minor suggestions, in particular on the issue that antero- or retrograde connections should be better specified in the text (see details below).

Minor issues:

Commissura is variously misspelled (Commisura, check)

Valvula is variously misspelled (Valbula, check)

McCormick, not Mccormick (check)

Abstract:

First line: Zebrafish .... rely or The zebrafish ... relies

Line 5: strongly bidirectionally connected

Line 8: ventral part (not parts)

Introduction:

Cathy McCormick's essential work on auditory connections in teleosts should be cited already in the main paragraph on page 3 prominently (ogether with Bass et al., 2000). This seminal work started in the 1980ties in the Northcutt laboratory.

Later same paragraph: Striedter (1991) also showed a TSc input to ATN in Ictalurus. Should be cited together with (Echteler, 1984; Northcutt, 2006).

Same paragraph later: Interestingly, AT receives input from the dorsomedial... (not: is connected)

Page 11:

The lateral lemnisci only carry fibers from somewhere. They do not project themselves to the TSc.

Next sentence: Projections to preoptic area and preglomerular area also arise from the TSc.

Discussion:

Line 3: anterior tuberal nucleus (dAT) is strongly reciprocally connected

Page 13, second paragraph: this is not the case in zebrafish (Yánez et al., 2022)

End of page 13: 3 citations: Bleckmann etc not listed in references)

Figure 2: Legend, line 4: labelling (red spots). Here three symbols in labeled nuclei need explanation: dots (retrograde), asterisks (anterograde?) and a third unidentifiable and unexplained element.

Reviewer 2

This is a well-designed study that identifies an anatomical connection between the torus semicircularis (TSc) midbrain auditory region and dorsal anterior tuberal nucleus (dAT) in zebrafish. The authors also use phosphorylated S6 protein (pS6) as an activity marker, describing the time course of pS6 expression onset and offset, and showing that both the TSc and dAT are activated by acoustic stimuli. I believe this research will be valuable to neuroscientists studying audition in fish, as well as anyone seeking to use pS6 as a neural activity marker in fish. However, I have one concern about the way pS6-labeled cells were counted, and some minor comments that I believe would improve the manuscript. My specific comments are below.

It is unclear why this approach to cell counting was taken. Specifically, I am concerned that the counting regions were determined based on areas that had highest pS6 expression in the region of interest. This may obscure differences between the groups. Ideally, pS6 expression would be quantified in the entire region of interest on the same number of sections from each animal. If a standardized counting region must be used, it should be applied to each section in a less biased way.

Minor comments:

Check to ensure that "acoustic" or "acoustical" are used consistently throughout

In Figure 2: Confocal images would make it much easier to distinguish cellular and fiber label. Especially for TSc and dAT images.

In Figure 2F: Please indicate what the arrows in F1 are pointing to in the figure caption

In the Discussion: "TSc was likely the main source of stimulation for the dAT as it strongly connects to this region" is unsupported. Despite the strong connection, the authors have not demonstrated it is the main source of stimulation. However, the pS6 expression seen in TSc at similar time points as dAT, and lack of activation in other regions, does suggest that the acoustic stimulus was the driver of pS6 expression in these regions

In the Discussion: "It is likely that the different time points for the peak responses of the two brain areas were due to statistical variability". The possibility that the results reflect a real difference in the time course of pS6 expression should be discussed.
